# Decreased Penetration Mechanism of Ranitidine Due to Application of Sodium Sulfobutyl Ether-β-Cyclodextrin

**DOI:** 10.3390/pharmaceutics15112593

**Published:** 2023-11-06

**Authors:** Rui Yang, Jing Zhang, Jiaqi Huang, Xiaofeng Wang, Huiying Yang, Qingri Jin

**Affiliations:** 1 NMPA Key Laboratory for Quality Research and Evaluation of Pharmaceutical Excipients, National Institutes for Food and Drug Control, Beijing 100050, China; yangr@nifdc.org.cn (R.Y.); zhangj20210422@126.com (J.Z.); 3322010400@stu.cpu.edu.cn (J.H.); wangxiaofeng@nifdc.org.cn (X.W.); 2 School of Pharmacy, Hangzhou Medical College, Hangzhou 311399, China

**Keywords:** sodium sulfobutyl ether-β-cyclodextrin, ranitidine, apparent permeability coefficient, permeability, inhibition

## Abstract

Permeability has an important effect on drug absorption. In this study, the effect of different concentrations of sodium sulfobutyl ether-β-cyclodextrin (SBE-β-CD) on the absorption of ranitidine was investigated to examine the mechanism of permeability changes. The results of a parallel artificial membrane permeability assay (PAMPA) showed that increasing the concentration of sodium sulfobutyl ether-β-cyclodextrin, 0, 0.12% (*w*/*v*), 0.36% (*w/v*) and 3.6% (*w/v*), respectively, caused the apparent permeability coefficient of ranitidine to decrease to 4.62 × 10^−5^, 4.5 × 10^−5^, 3.61 × 10^−5^ and 1.08 × 10^−5^ in Caco-2 cells, respectively. The same results were obtained from an oral pharmacokinetic study in rats. Further studies indicated that SBE-β-CD significantly increased the zeta potential of ranitidine. SBE-β-CD interacted with ranitidine charges to form a complex that reduced ranitidine permeability, and SBE-β-CD should be chosen with caution for drugs with poor permeability.

## 1. Introduction

According to the International Coordination Committee for the Registration of Pharmaceutical Products for Human Use (ICH) guidelines and the M9 Bioequivalence Exemptions Based on the Biopharmaceutical Classification System [1,2,3,4] permeability is closely related to the rate and extent of drug absorption in the body and is one of the key factors in the transport of drugs in the body through expanded membranes and is relevant to drug development. Pharmaceutical excipients are non-physiologically active substances in pharmaceuticals that affect their absorption and bioavailability in the body [5,6,7]. For poorly permeable drugs, permeation enhancers are commonly used [8,9,10,11], for example, fatty alcohols, which increase drug permeability by altering the structure and properties of biofilms. Co-solvents could provide a way to address the issue of insoluble drugs, but they also lead to change in permeability [12]. 

SBE-β-CD [13,14,15,16,17,18], a new pharmaceutical excipient often used as a co-solvent, has been included in USP44-NF39 under the name Betadex Sulfobutyl Ether Sodium, and is currently available as Captisol^®^ [19,20] and Dexolve^®^ [21]. It is widely used in biologics and insoluble drugs [22,23,24]. With an average degree of substitution of 6.5, sulfobutyl can be substituted at the 2-, 3- and 6-hydroxyl groups of the β-glucose unit, ameliorating the nephrotoxicity problems of β-cyclodextrins [25], which is a better co-solvent for solubility. However, usually, the increase in drug solubility with co-solvents is followed by a corresponding change in drug permeability, and there is a delicate balance between the two effects [12,26,27,28]. Under certain pH conditions, when the osmotic effect of increased solubility exceeds the inhibition of permeability by the co-solvent, an increase in drug permeation is presented, e.g., hydroxypropyl-β-cyclodextrin (HP-β-CD) at pH 6.2 increases the permeability of albendazole; conversely, the permeation of the drug is reduced, e.g., HP-β-CD at pH 6.2 reduces the permeability of bupivacaine. For drugs with good solubility, cyclodextrins inhibit their permeability in vivo [29]. Current studies on SBE-β-CD have focused on the effect on solubility, with less exploration of permeability and a lack of further studies of the mechanisms, both of which play a pivotal role in drug absorption. It is important to investigate the mechanisms through which SBE-β-CD affects drug permeability in vitro and in vivo based on the fully dissolved state of the drug. To elucidate the mechanism by which SBE-β-CD affects drug permeability, ranitidine was chosen as a model drug to study the changes in permeability under human intestinal pH conditions. As a histamine-like H2 receptor blocker [30], ranitidine is commonly used in the treatment of peptic gastric ulcer and acid reflux; the drug is readily soluble in water, which precludes osmotic effects due to increased solubility.

**Figure 1:**
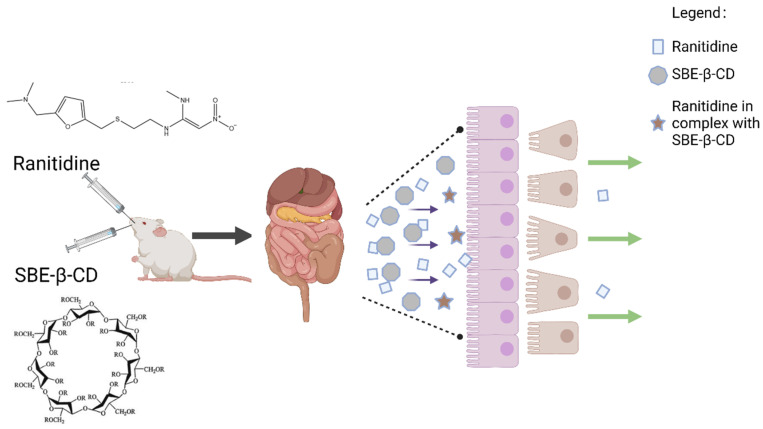
SBE-β-CD and ranitidine form a complex to reduce permeability.

In this study, the effects of different concentrations of SBE-β-CD on the permeability of ranitidine and its absorption in rats were investigated using parallel artificial membrane permeability assay (PAMPA), a permeability study on Caco-2 cells, and pharmacokinetic studies in rats with oral administration of ranitidine and SBE-β-CD. Based on this finding, a zeta potential study was then designed to investigate the mechanism through which SBE-β-CD reduces drug permeability (Figure 1).

## 2. Materials and Methods

### 2.1. Materials

#### 2.1.1. Reagents

Sodium sulfobutyl ether-β-cyclodextrin (Shanghai, China; manufacturer EC0602202107757), ranitidine (Jiangxi, China; RWE20220520, 99.5%) and ranitidine hydrochloride standard (Beijing, China; lot 100163, 99.9%) were purchased from China Institute for Food and Drug Control. PBS (0.01 M pH 7.2–7.4) was obtained from Solarbio company (Beijing, China). Anhydrous potassium dihydrogen phosphate and dodecahydrate and disodium hydrogen phosphate were purchased from Sinopharm Key Chemical Reagent Co., Ltd., (Shanghai, China). Acetonitrile was obtained from Amethyst company (Beijing, China). Human colon cancer cells Caco-2 cells were purchased from ATCC.

#### 2.1.2. Animals

Twenty healthy SD rats of similar body weight were purchased from the Experimental Animal Centre of Hangzhou Medical College, Zhejiang Province. After 1 week of acclimatization in the experimental animal room with a constant temperature and humidity, the rats were randomly divided into the ranitidine group, the 0.12% SBE-β-CD and ranitidine group, the 0.36% SBE-β-CD and ranitidine group, and the 3.6% SBE-β-CD and ranitidine group.

### 2.2. Methods

#### 2.2.1. Preparation of Working Solutions

(1) Preparation of donor chamber buffers

An amount of 1.7 g of potassium dihydrogen phosphate and 1.775 g of anhydrous disodium hydrogen phosphate were added into 1000 mL of purified water, vortex-mixed and dissolved using ultrasonication and the pH was adjusted to 6.8.

(2) Preparation of reception chamber buffers

An amount of 1.7 g of potassium dihydrogen phosphate and 1.775 g of anhydrous disodium hydrogen phosphate were added into 1000 mL of purified water, stirred, dissolved using ultrasonication and the pH was adjusted to 7.4.

(3) Preparation of working solutions in the donor room

The appropriate amount of ranitidine hydrochloride was weighed and fixed to 25 mL of donor chamber buffer and prepared as a working solution at a concentration of 2 mg/mL. The mother liquor, 1.5 mL, was fixed to 5 mL of donor chamber buffer to obtain 600 μg/mL of ranitidine hydrochloride solution. Amounts of 12 mg, 36 mg and 360 mg SBE-β-CD were accurately weighed in 10 mL volumetric flasks and dissolved with a small amount of donor chamber buffer. Then 3 mL of ranitidine hydrochloride working solution was added and the volume was filled with buffer up to 10 mL. Three drug solutions containing different concentrations of SBE-β-CD were prepared.

#### 2.2.2. Parallel Artificial Membrane Permeability Assay

(1) Measurement of Ranitidine concentration

A reverse column (SunFireTM C18, 4.6 × 150 mm, 5 μm) was used. The mobile phase was phosphate buffer solution and acetonitrile in a ratio of 1:1. The column temperature was set to 30 °C; the flow rate was set to 1 mL/min; the injection volume was set to 10 μL; the detection wavelength was set to 314 nm; the detector was a high performance liquid phase variable wavelength detector (Agilent). An amount of 0.1 g of ranitidine hydrochloride was added into a 100 mL flask; then 1 mL of 50% sodium hydroxide solution was added, followed by water and fill up to volume and it was vortex-mixed. The system suitability test solution was obtained and 10 μL was injected into liquid chromatography for system suitability detection.

(2) Two-way conversion experiment

Three drug solutions containing different concentrations of SBE-β-CD and 400 μL of ranitidine hydrochloride solution were taken. Each was placed in the donor chamber of the PermeaPad Plate, and three parallel experiments were performed for each set of solutions. After placing the receiving chamber above the donor chamber, 200 μL of receiving chamber buffer was added to the receiving chamber. A timer was started, and at 2, 4, 8 and 12 h after the start of the experiment, all the receiver chamber solutions were removed and new 200 μL of PBS buffer was added. The ranitidine content of the removed samples was determined. The apparent osmolality Papp value was calculated for each solution.

Apparent permeability coefficient equation was as follows: $Papp=\frac{dC}{dt}\cdot \frac{Vr}{A\cdot C_{0}}$, Vr is the volume, dC/dt is the concentration of drug permeated per unit time, A is the area of the membrane, C_0_ is the initial drug concentration.

#### 2.2.3. Caco-2 Cell Experiment

(1) Determination of ranitidine concentration using UPLC/MS/MS

To accurately measure the concentration of ranitidine in rat plasma, we used an ultra-performance liquid chromatography (UPLC) mass spectrometer (MS) for the analysis of plasma samples. The mass spectrometry analysis was performed using an electrospray ion source (ESI), scanned in positive ion (ES+) mode and detected in multiple ion reaction monitoring (MRM) mode. The mass-to-charge ratio (m/z) of the ranitidine parent ion was set to 315 and the mass-to-charge ratios of the secondary mass spectrometry daughter ion were set to 176 and 130, with corresponding collision voltages of 23 eV and 37 eV. Other parameters were set as follows. Desolventizing temperature was set to 500 °C, desolventizing gas volume was set to 800 L/h, cone hole voltage was set to 25 V, cone hole gas flow rate was set to 50 L/h and capillary voltage was set to 3.50 kV.

(2) preparation of working solution for caco-2 cell experiment

An appropriate amount of ranitidine hydrochloride was taken, weighed accurately, and the volume was fixed to 25 mL with 37 °C Hank’s buffer. A master batch was prepared at a concentration of 2 mg/mL. An amount of 1.5 mL of working solution was added to 5 mL of Hank’s buffer at 37 °C to obtain 600 μg/mL of the test drug solution. SBE-β-CD 12 mg, 36 mg and 360 mg were weighed precisely, dissolved with a small amount of 37 °C Hank’s buffer, 3 mL of ranitidine hydrochloride mother liquor was added and the volume was fixed to 10 mL with 37 °C Hank’s buffer to obtain drug solutions with excipient concentrations of 0.12% (*w/v*), 0.36% (*w/v*) and 3.6% (*w/v*), respectively.

(3) Caco-2 cell culture

Caco-2 cells were cultured and the TEER values on both sides of the cells were measured. After the TEER values reached 300 Ω-cm^2^, the 6-well Transwell cell culture plates were washed twice with pre-warmed HBSS on both sides and fluorescent yellow (20 μg/mL) was added on the AP side and HBSS on the BL side, and then incubated at 37 °C and 5% CO_2_ in a saturated humidity incubator for 1 h. The fluorescent yellow concentration and (apparent permeability coefficient) Papp were calculated at 30 and 60 min, respectively. If the leakage of fluorescent yellow is <5% and Papp ≤ 5 × 10^−7^ cm/s, then the Caco-2 cell monolayer is intact and can be used for drug permeation assay.

(4) Measurement of transepithelial transport of ranitidine

An amount of 1.5 mL of the test drug solution and 2.6 mL of the blank solution were precisely measured and placed on the AP side and BL side, respectively, and the positions of the test drug solution and the blank solution were switched to verify the transfer in the opposite direction. At 15, 60 and 120 min after the start of the experiment, 300 μL of each sample was taken and supplemented with 300 μL of blank buffer. Four sets of parallel experiments were performed for each set of solutions along with a blank control experiment.

#### 2.2.4. Pharmacokinetic Study of Ranitidine in Rats

(1) Determination of ranitidine concentration in rat plasma

The determination method is described in Section 2.2.3.

(2) Pharmacokinetic study of ranitidine in rats

Anesthesia was administered intramuscularly using tiletamine hydrochloride and Zolazepam at a dose of 50 mg/kg while the femoral artery and vein of the rats were cannulated with PE-50 polyethylene tubing and body temperature was maintained with a heat lamp. The animals were divided into three groups. The first group received ranitidine at a dose of 13.5 mg/kg by oral administration. The second group received oral administration of ranitidine and SBE-β-CD at doses of 13.5 mg/kg and 270 mg/kg, respectively. The third group received oral administration of 13.5 mg/kg and 810 mg/kg of ranitidine and SBE-β-CD, respectively. Blood samples measuring 300 μL were collected from the femoral artery at 0, 0.25, 0.5, 1, 2, 2.5, 3, 4, 6, 8, 10 and 12 h. An equal volume of saline was used instead of blood volume to compensate for fluid loss. The area under the plasma concentration–time curve was calculated from zero to infinity (AUC) using a trapezoidal extrapolation method. For extrapolation, the area from the last data point to time infinity was estimated from the end rate constant. Time-averaged overall clearance (CL) and steady-state apparent volume of distribution (Vz) were calculated with non-compartmental analysis using WinNonlin^®^ (version 3.1, Pharsight, Mountain View, CA, USA) standard methods.

#### 2.2.5. Zeta Potential Determination

Ranitidine hydrochloride solution, 0.06%, 0.12%, 0.24%, 0.36%, 0.72%, 1.44% and 3.6% SBE-β-CD solutions and a mixture of SBE-β-CD and ranitidine at the same concentrations were taken, and potentiometric measurements were performed using a Malvern particle size potentiometer.

## 3. Results

### 3.1. In Vitro Analysis of Inhibitory Effect of Ranitidine by SBE-β-CD

#### 3.1.1. Parallel Artificial Membrane Permeability Assay

(1) Measurement of Ranitidine concentration

The retention time of ranitidine was 2.1 min, and the separation from the main impurities produced by base destruction was 9. When the concentration of ranitidine was in the range of 5~500 μg/mL, the drug content showed a good linear relationship with the chromatographic peak area (R^2^ = 0.999), and the limit of detection was 0.1 μg/mL and the limit of quantification was 0.25 μg/mL. The RSD of the peak area of the test solution was 0.11% after 6 consecutive injections, indicating that the precision was virtuous. The RSD of the peak area of the test solution was 0.28% after 0, 2, 4, 6, 8 and 10 h of preparation, representing that the stability of the solution was excellent.

(2) Measurement of transepithelial transport of ranitidine by Pampa assay

**Figure 2:**
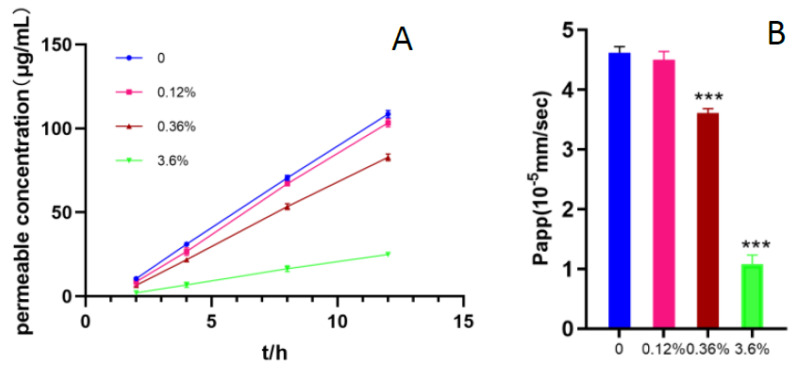
Effect of different concentrations of SBE-β-CD on ranitidine permeability in PAMPA test. (**A**): Different concentrations of SBE-β-CD on ranitidine permeability, (**B**): the ANOVA test of ranitidine Papp value. *** *p* < 0.001.

The Permea Pad Plate 96-well permeation plate was used to investigate the effect of different concentrations of SBE-β-CD on the permeability of ranitidine. In Figure 2, it can be seen that as the concentration of SBE-β-CD increased to 0.36% (*w/v*), it produced an inhibitory effect on the permeation of ranitidine. Further increases in SBE-β-CD concentration increased the inhibition of drug permeability.

Using the apparent coefficient of permeability equation: $\mathrm{Papp}=\frac{\mathrm{dC}}{\mathrm{dt}}\cdot \frac{\mathrm{Vr}}{A\cdot C_{0}}$, Vr is the volume; dC/dt is the concentration of drug permeated per unit time; A is the area of the membrane; C_0_ is the initial drug concentration. The apparent permeability of ranitidine at different concentrations of SBE-β-CD was calculated to be 4.62 × 10^−5^; the apparent permeability of ranitidine at 0.12% (*w/v*) SBE-β-CD was 4.50 × 10^−5^; at 3.6% (*w/v*) SBE-β-CD the ranitidine Papp value was 1.08 × 10^−5^. The calculation of Papp values showed that with the change in concentration, SBE-β-CD exerted different degrees of inhibition on the penetration of ranitidine.

#### 3.1.2. Results of Transepithelial Transport of Ranitidine by Caco-2 Cells

**Figure 3:**
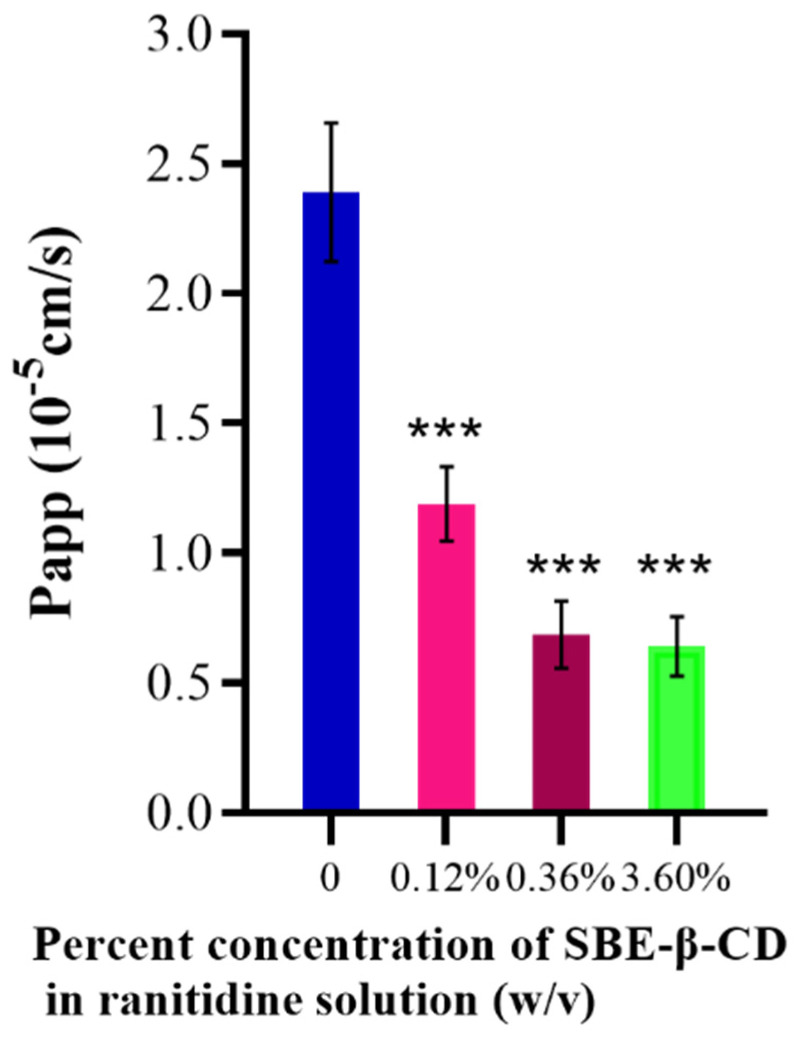
Effect of different concentrations of SBE-β-CD on ranitidine permeability in Caco-2 cell analysis. *** *p* < 0.001.

The effect of different concentrations of SBE-β-CD on the transport of ranitidine was investigated using Caco-2 cell culture in 8-well transwell plates. Using the apparent osmolality formula, the Papp value of ranitidine was calculated to be (2.39 ± 0.267) × 10^−5^. The apparent Papp value of ranitidine under the effect of different concentrations of SBE-β-CD was calculated to be: 1.19 × 10^−5^ under the effect of 0.12% (*w/v*) SBE-β-CD; under the effect of 0.36% (*w/v*) SBE-β-CD, the ranitidine Papp value was 6.87 × 10^−6^; and the ranitidine Papp value was 6.42 × 10^−6^ at a dose of 3.6% (*w/v*) SBE-β-CD (Table 1). The Papp value of ranitidine with the addition of a certain concentration of SBE-β-CD was significantly different from the Papp value of ranitidine (Table 1 and Figure 3).

### 3.2. In Vivo Analysis of Inhibitory Effect of Ranitidine by SBE-β-CD

**Figure 4:**
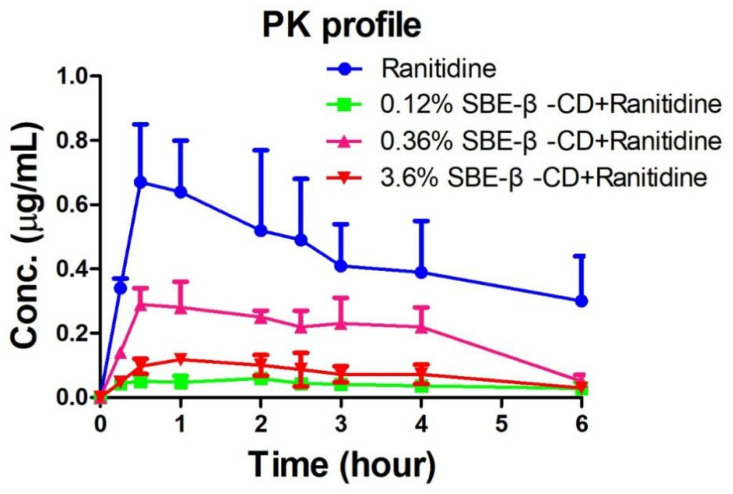
Ranitidine plasma concentration–time curve in rats.

The peak areas of ranitidine obtained from the rat plasma samples were determined and brought into the regression equation to calculate the concentration of ranitidine in plasma samples. Table 2 shows the pharmacokinetic parameters detected in rats after the administration of ranitidine and SBE-β-CD at a dose of 0.12% and 0.36%. Pharmacokinetic parameters such as elimination half-life (t_1/2_), time to peak (T_max_), peak concentration (C_max_), area under the drug–time curve (AUC_all_), apparent volume of distribution (Vz), body clearance (CL), and mean residence time (MRT_last)_ were calculated using the non-compartment model of Winnonlin software 5.0 after administration of 0.12% SBE-β-CD and 0.36% SBE-β-CD with ranitidine. The peak concentration of ranitidine after SBE-β-CD was significantly lower than that of ranitidine API (0.73 ± 0.18 μg/mL vs. 0.064 ± 0.009 μg/mL, 0.30 ± 0.05 μg/mL, 0.13 ± 0.007), indicating that both the rate and extent of absorption of ranitidine were influenced by SBE-β-CD (Figure 4). The area under the drug–time curve of ranitidine after injection of 0.12% SBE-β-CD and 0.36% SBE-β-CD was also significantly reduced compared to that of ranitidine only (3.6 ± 1.2 h μg/mL vs. 0.3 ± 0.1 h·μg/mL, 0.5 ± 0.1 h·μg/mL). This result may be due to SBE-β-CD affecting the rate and extent of absorption of ranitidine, resulting in a decline in the amount of drug reaching the systemic circulation.

### 3.3. Mechanism of Inhibition of Ranitidine Permeability In Vivo by SBE-β-CD

**Figure 5:**
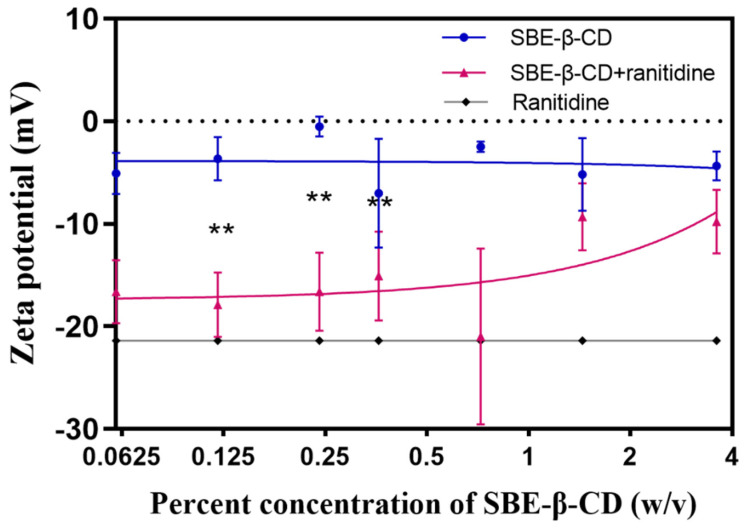
The zeta potential curves of different concentrations of SBE-β-CD solutions and mixed solutions of SBE-β-CD and ranitidine. ** *p* < 0.01.

To investigate the mechanism through which SBE-β-CD affects the permeability of ranitidine, zeta potential measurements were performed on ranitidine hydrochloride solution, 0.06%, 0.12%, 0.24%, 0.36%, 0.72%, 1.44% and 3.6% of SBE-β-CD solution, and the corresponding concentrations of the mixture of SBE-β-CD and ranitidine. The experimental results (see Table 3 and Figure 5) showed that the zeta potential of the mixed solution of SBE-β-CD with ranitidine increased with increasing concentrations of SBE-β-CD. The zeta potential of the 0.06% SBE-β-CD and ranitidine mixture was 16.60 mV, which was significantly lower than the 0.06% SBE-β-CD solution (−5.09 mV). The zeta potential of the 0.12% SBE-β-CD and ranitidine mixture was −17.90 mV, which was significantly lower than that of the 0.12% SBE- β-CD solution (−3.66 mV). The zeta potential of 0.24% SBE-β-CD and ranitidine mixture was −16.6 mV, which was lower and significantly different from that of 0.24% SBE-β-CD solution (−0.52 mV). Continuing to increase the concentration of SBE-β-CD in ranitidine solution concentration, the zeta potential gradually approached that of the SBE-β-CD solution, probably due to the excess of SBE-β-CD after increasing the concentration, consequential in the dominance of the potential of SBE-β-CD in the solution. Using Pearson correlation analysis, the Zeta potential was positively and strongly correlated with the concentration of SBE-β-CD in the ranitidine mixture (r = 0.7282, *p* = 0.0405).

Zeta potential is a measure of the stability of the dispersion system, and changes in this indicator suggest the formation of complexes in the dispersion system. The interaction between SBE-β-CD and ranitidine consists of commonly attractive charge interactions. Additionally, the sulphury element in SBE-β-CD and the nitrogen element in ranitidine readily form hydrogen bonds, causing them to bind and form complexes. In the human intestine there is an unstirred aqueous layer consisting of in vivo mucus with a thickness of 30–100 μm [25]. Once formed, the complexes need to pass successively through the hydrated layer and the lipid barrier before they can enter the body. The change in size and shape causes the complex to encounter greater spatial site resistance when passing through the unstirred aqueous layer and biofilm, resulting in slower absorption and reduced permeability of ranitidine.

In summary, the charge interaction between SBE-β-CD and ranitidine occasioned the formation of a complex, which in turn altered the rate of absorption and permeability of ranitidine. This interaction may have influenced the behavior of the drug as it passed through the hydrated layer and biofilm by increasing the spatial site resistance. This has important implications for understanding the mechanisms through which SBE-β-CD affects drug permeability and provides a useful reference for drug design and optimizing drug delivery systems.

## 4. Discussion

Drug permeability is one of great factors for drug development and therapeutic efficacy, as it directly affects the bioavailability, pharmacodynamic properties and therapeutic efficacy of the drug. Drug permeability is influenced by many factors, such as particle size [31]. SBE-β-CD is often used as a co-solvent and excipient in pharmaceutical formulations, and the mechanisms through which this excipient affects drug permeability are less well considered. Therefore, in this study, two in vitro permeation systems, PAMPA assay and Caco-2 cell assay, were applied to investigate the effect of SBE-β-CD on the permeability of ranitidine. After that, pharmacokinetic experiments in rats were carried out to further confirm that SBE-β-CD could inhibit the permeability of ranitidine in vivo. The mechanism is related to the formation of the complex, with changes in size and shape leading to increased spatial site resistance to passage through the intestinal barrier, which in turn leads to slower absorption and reduced permeability.

For drugs with poor oral permeability, the correct choice of drug excipient is crucial to improve the bioavailability and efficacy of the drug. This study investigates the mechanism of SBE-β-CD inhibition of drug permeability and fills a gap in this research field. Our results provide an important reference for the prescription design of drugs with poor oral permeability and are of great significance. For drugs with poor oral permeability, changes in permeability can affect the acquisition of biological immunity [32], so the dose of SBE-β-CD should be carefully selected. Further studies could explore the mechanism of the effect of other cyclodextrin polymers on drug permeability. Cyclodextrin polymers [33] are a class of substances based on the modification of their substituents by the cyclodextrin structure, which have similar cavity structures and molecular recognition capabilities, but may differ in their chemical structure and properties. By studying the interaction of different types of cyclodextrin polymers with drugs, a more comprehensive understanding of the effects of these excipients on drug permeability can be obtained and can provide a theoretical basis for the development of novel cyclodextrin excipients. In addition, the effect of the application of SBE-β-CD in combination with other excipients on drug permeability can be further investigated. The selection and combination of excipients may have a synergistic effect on drug permeability, which helps to improve the bioavailability and efficacy of the drug. By studying the combination and ratio of different excipients, the permeability and therapeutic effect of the drug can be optimized and the absorption and bioavailability of the orally administered drug can be improved.

## 5. Conclusions

In this study, ranitidine was selected as a model drug, and its permeability was investigated in vitro and in vivo with SBE-β-CD at human intestinal pH, and the mechanism through which SBE-β-CD affects drug permeability in vivo was investigated based on the complete dissolution of the drug. SBE-β-CD was found to inhibit the in vivo permeability of ranitidine under human intestinal pH conditions with a mechanism related to the formation of complexes, where the charge of SBE-β-CD interacts with ranitidine to form complexes that are subject to greater resistance to crossing the physiological barrier, resulting in changes in drug permeability. Consequently, the dose of SBE-β-CD should be carefully selected when applying SBE-β-CD for the oral administration of drugs with poor permeability.

## Figures and Tables

**Table 1:** Papp value for Caco-2 cell model experiments.

	Papp (A-B, cm/s)	Papp (A-B, Mean ± SD, *n* = 4)
A-B	2.1760 × 10^−5^	(2.39 ± 0.267) × 10^−5^
	2.8514 × 10^−5^	
	2.2886 × 10^−5^	
	2.2605 × 10^−5^	
B-A	2.60281 × 10^−5^	(2.60 ± 0.152) × 10^−5^
	2.4513 × 10^−5^	
	2.84091 × 10^−5^	
	2.48918 × 10^−5^	
0.12%	1.33189 × 10^−5^	(1.19 ± 0.143) × 10^−5^
	1.20058 × 10^−5^	
	1.13492 × 10^−5^	
	1.07864 × 10^−5^	
0.36%	8.16017 × 10^−6^	(6.87 ± 1.30) × 10^−6^
	4.87734 × 10^−6^	
	6.56566 × 10^−6^	
	7.87879 × 10^−6^	
3.6%	7.50361 × 10^−6^	(6.42 ± 1.15) × 10^−6^
	4.97114 × 10^−6^	
	5.62771 × 10^−6^	
	7.59740 × 10^−6^	

**Table 2:** Pharmacokinetic parameters of ranitidine at a dose of 13.5 mg/kg by oral administration.

PK Parameters	Unit	Ranitidine Only	Ranitidine and 0.12% SBE-β-CD	Ranitidine and 0.36% SBE-β-CD	Ranitidine and 3.6% SBE-β-CD
T_max_	h	0.83 ± 0.29	1.17 ± 0.76	1.33 ± 0.58	1.50 ± 0.87
C_max_	μg/mL	0.73 ± 0.18	0.064 ± 0.009 *	0.30 ± 0.05 *	0.13 ± 0.007 *
t_1/2_	h	6 ± 6	5 ± 2	1 ± 0	2 ± 1
AUC_all_	h·μg/mL	3.6 ± 1.2	0.3 ± 0.1 *	1.3 ± 0.2	0.5 ± 0.1 *
AUC__Extrap_	%	27 ± 30	31 ± 19	4 ± 3	18 ± 12
Vz	L/kg	19.8 ± 10.8	191 ± 17 ***	20.5 ± 8.8	82.0 ± 24.6 *
CL	L/h/kg	3.07 ± 1.84	35.4 ± 20.5	10.3 ± 1.9 *	27.2 ± 12.4
AUMC_last_	h·h·μg/mL	14.1 ± 5.5	1.0 ± 0.4	3.6 ± 0.5	1.1 ± 0.4 *
MRT_last_	h	4 ± 1	3 ± 0	3 ± 0	3 ± 0

* *p* < 0.05; *** *p* < 0.001.

**Table 3:** Zeta potential of SBE-β-CD with different concentrations.

	Zeta Potential (mV)	*p*-Value of Levene’s Test for Equality of Variances	*p*-Value of *T* Test for Equality of Means
Ranitidine	−21.40 ± 6.90	/	/
0.06% SBE-β-CD	−5.09 ± 2.02	0.5988	0.0057 **
0.06% SBE-β-CD + Ranitidine	−16.60 ± 3.09		
0.12% SBE-β-CD	−3.66 ± 2.12	0.6235	0.0029 **
0.12% SBE-β-CD + Ranitidine	−17.90 ± 3.15		
0.24% SBE-β-CD	−0.52 ± −0.52	0.1210	0.0021 **
0.24% SBE-β-CD + Ranitidine	−16.6 ± −16.6		
0.36% SBE-β-CD	−7.02 ± 5.29	0.8090	0.1108
0.36% SBE-β-CD + Ranitidine	−15.10 ± 4.36		
0.72% SBE-β-CD	−2.50 ± 0.53	0.0076	0.0203
0.72% SBE-β-CD + Ranitidine	−21.00 ± 8.58		
1.44% SBE-β-CD	−5.20 ± 3.55	0.9211	0.2148
1.44% SBE-β-CD + Ranitidine	−9.31 ± 3.28		
3.6% SBE-β-CD	−4.36 ± 1.41	0.3447	0.0504
3.6% SBE-β-CD + Ranitidine	−9.79 ± 3.09		

** *p* < 0.001.

## Data Availability

Not applicable.
